# The risk of acute and early HIV (AEH) infection among MSM with different behaviour trajectories: an open cohort study in Tianjin, China, 2011–2019

**DOI:** 10.1186/s12879-023-08001-9

**Published:** 2023-01-20

**Authors:** Xiaomeng Wang, Tiantian Zhang, Qinxue Chang, Chun Wang, Keyun Wang, Zeyang Yu, Honglu Zhang, Huijie Huang, Desheng Song, Elissa Peixoto, Jie Yang, Changping Li, Zhuang Cui, Yuanyuan Liu, Jun Ma

**Affiliations:** 1 Department of Epidemiology and Biostatistics, Tianjin Medical University, 22, Qixiangtai Road, Heping District, Tianjin, 300070 China; 2 Medical Service Division, Institute of Hematology and Blood Diseases Hospital, Chinese Academy of Medical Sciences & Peking Union Medical College, Tianjin, China; 3 “Shenlan” Public Health Counseling Service Center, Tianjin, China

**Keywords:** Acute and early HIV, Sexual risk behaviour trajectory, MSM, Voluntary HIV counselling and testing, Group-based trajectory model, Open cohort study

## Abstract

**Background:**

Acute and early HIV (AEH) infection is characterized by a high viral load and infectivity. Approximately 50% of cases of HIV-1 transmission occur during AEH. Understanding sexual behaviour trajectories would be useful for predicting changes in the risk of HIV acquisition. However, few studies have investigated sexual behaviour trajectories and their association with AEH acquisition. This study identified behaviour trajectories among men who have sex with men (MSM), determined the risk of AEH infection, and compared risk factors between different behaviour trajectories.

**Methods:**

The study was based on an ongoing prospective open cohort of voluntary HIV counselling and testing (VHCT) among MSM in Tianjin, China. From 2011 to 2019, 1974 MSM were recruited. Group-based trajectory modelling (GBTM) was used to identify behaviour trajectories by constructing a sexual risk behaviour score. Logistic regression and generalized estimating equation (GEE) were used to compare the risk of AEH infection and risk factors for different behaviour trajectories. All data analyses were performed using SAS 9.4.

**Results:**

The incidence of AEH infection was 1.76/100 person-years, with 64 AEH infections documented in 3633 person-years of follow-up. Three sexual behaviour trajectories were identified: CL (consistently low risk, 35.46%), CH (consistently high risk, 42.71%) and HTL (high to low risk, 21.83%). MSM in the HTL and CH groups had higher AEH infection rates than MSM in the CL group (6.73%, 3.08% and 1.28%, respectively), with ORs of 5.54 (2.60, 11.82) and 2.44 (1.14, 5.25), respectively. MSM aged 30–50 years old and MSM who underwent HIV testing in the last year were more likely to be in the CH group and HTL group. In addition, the HTL group was characterized by a lower likelihood of local registration and a higher likelihood of working as a MSW.

**Conclusion:**

MSM in the CH group and the HTL group had a higher risk of AEH infection. In the future, VHCT should be performed more often among younger MSM, and HIV counselling should be given the same priority as HIV testing. In addition, VHCT combined with PrEP may have a better preventive impact on MSM with a high risk of AEH infection.

**Supplementary Information:**

The online version contains supplementary material available at 10.1186/s12879-023-08001-9.

## Background

In China, men who have sex with men (MSM) are the only subpopulation with an increasing rate of HIV infection [1]. An important driver of the ongoing HIV epidemic among MSM could be acute and early HIV (AEH) infection [2]. AEH infection is characterized by a high viral load and infectivity and has contributed disproportionately to HIV transmission [3]. A previous study showed that approximately 50% of cases of HIV-1 transmission occur during AEH [4]. Early detection of HIV is essential to reduce the viral load and HIV transmission by starting antiretroviral therapy (ART) and limiting risky sexual behaviours.

Previous studies have proven that different factors contribute to the high risk of HIV transmission among MSM, such as unprotected anal intercourse, casual sexual partners, multiple sexual partners [5], recreational drug use [6], and insufficient use of preexposure prophylaxis (PrEP) and postexposure prophylaxis (PEP) [7, 8]. However, sexual behaviours are not static over the lifetime. Identifying how sexual behaviour changes over time is necessary to implement more precise HIV prevention interventions.

Changes in sexual behaviour over time are different for each MSM, but the trajectories of behaviour change at the group level may be universal. Heather et al. identified three trajectories among MSM using a predefined sexual risk behaviour score: low-risk, medium-risk, and high-risk groups [9]. A similar study based on Amsterdam cohort studies (ACSs) also found three trajectories: low risk, falling high risk and rising high risk [10]. With respect to inconsistent condom use during receptive anal sex in West Africa, Coulaud et al. posited two trajectories: medium-risk exposure and high-risk exposure [11]. Understanding sexual risk behaviour trajectories would be useful for predicting changes in the risk of HIV acquisition and transmission. In 2021, a study in China found that MSM with a behaviour pattern of “consistent or develop risky profile” had an increasing likelihood of AEH infection compared with MSM with a “maintain/develop safety profile” [12]. At present, few studies have investigated sexual behaviour trajectories and the association with AEH acquisition.

Based on an 8-year voluntary HIV counselling and testing (VHCT) project conducted in Tianjin, China, between 2011 and 2019 among MSM, we aimed to (1) identify sexual risk behaviour trajectories using a comprehensive sexual risk behaviour score; (2) compare the AEH infection risk between different behaviour trajectories; and (3) determine the factors associated with a high AEH infection risk trajectory.

## Methods

### Study design

Our study is an ongoing prospective open cohort of VHCT among MSM in Tianjin, China. The VHCT project was conducted at the “Shenlan” community-based organization (CBO) and at venues where MSM gather (e.g., bathhouses, bars) and was implemented by professional staff at the CBO who were also MSM. All participants completed a standard questionnaire during HIV counselling to collect information about their demographics, sexual risk behaviour and history of health services. Each participant underwent a rapid HIV antibody test and an amplified nucleic acid test for HIV confirmation. During the 20 min waiting for the rapid test results, all participants were provided condoms and lubricants as compensation for their cooperation and were provided sex education and advice based on the responses on their questionnaires. The results of the amplified nucleic acid test took 1 week to come in, and all participants were informed of their results. Participants were recommended to return for HCT every 3 months. However, HCT was provided whenever MSM wanted an HIV test due to the high-risk sexual behaviour of this population.

### Study participants

Eligible participants included MSM who were HIV-negative at enrolment and had a history of at least two visits. For individuals living with HIV at follow-up, only MSM diagnosed with AEH infection were included. Of the 6565 MSM enrolled in the study from October 2011 to December 2019, 6133 MSM were excluded from the present study (432 were diagnosed with HIV at enrolment, 4096 had only one visit, and 63 had chronic HIV infection). Finally, a total of 1974 MSM were included (Additional file 3: Fig. S1). The average follow-up time was 1.84 person-years.

### Measures

Demographic data included age (< 30, 30–49 and ≥ 50), marital status (married or not married), household registration location (local or nonlocal) and time of local residence (≤ 1 year or > 1 year).

The following sexual risk behaviours were assessed: working as a male sex worker (MSW) (yes or no), history of anal sex (yes or no) within the previous 6 months, number of instances of anal sex within the previous 7 days, condom use during last sexual intercourse with a man (yes or no), commercial sexual behaviour (yes or no), multiple sexual partners (yes or no) and number of anal sex partners within the previous 6 months.

If participants accepted HIV risk reduction education or condom/lubricant distribution from trained peers and institutions (e.g., CBO, Centers for Disease Control (CDC) and hospitals) in the last year, they were considered to have received health services. We also assessed whether participants underwent HIV testing in the last year (yes or no).

The sexual risk behaviour score was defined as follows. Four behaviours considered to be associated with significant HIV infection in previous literature [10, 13, 14] were used to construct sexual risk behaviour scores: condom use during last time engaging in anal sex with a man within the previous 6 months, frequency of condom use within the previous 6 months, number of times engaging in anal sex within the previous 7 days and number of sexual partners within the previous 6 months. Variable assignments are displayed in detail in Additional file 1: Table S1. The sexual risk behaviour score was calculated for each participant at each HCT visit. Scores ranged from 0 to 6, and higher scores indicated more risky sexual behaviour.

AEH infection was defined according to the following criteria: (1) HIV-1 RNA level of ≥ 1000 copies/mL for two nucleic acid amplification tests (NAAT, Roche COBAS TAQMAN48) in the following situations [15]: (a) negative fourth-generation Ab/Ag screening test (SB rapid test, HIV Ag/Ab combo, Alere, CFDA registered) result but positive HIV pooled PCR result; (b) positive fourth-generation Ab/Ag screening test result and negative/indeterminate ELISA (Wantai Biological Pharmaceutical Co., Ltd, Beijing, China) test result; (c) negative or indeterminate Western blot (WB, MP Biomedical Asia Pacific Pte Ltd, Singapore) test result; (2) a positive HIV-1 antibody test result and a documented negative HIV-1 antibody test result within the previous 6 months [16].

### Data analyses

#### Group-based trajectory modelling (GBTM)

Across the follow-up period, group-based trajectory modelling (GBTM) was used to identify sexual behaviour trajectories by sexual risk behaviour score. One of the main advantages of this method is that the missing values are considered; therefore, we can include participants with at least two follow-up visits. The PROC TRAJ SAS procedure was used to calculate the probability of each participant belonging to each trajectory group, and individuals were assigned to trajectories based on their highest probability of trajectory membership. By fitting a series of models with three to five trajectories, several models were constructed assuming linear, quadratic, and cubic shapes of the trajectory group curves. In determining model fit and the optimal number of trajectory groups, model fit statistics and interpretability were considered. Statistics included the Bayesian information criterion (BIC), Akaike information criterion (AIC) and significance of the shape of trajectory group curves. The model fitting process started with a cubic specification for the shape of trajectory group curves, and then, significant terms were assessed [17–19]. After excluding nonsignificant terms, the trajectory models were refitted again. The models used a zero-inflated Poisson distribution to account for the large number of participants who reported having no risky sexual behaviours.

#### Logistic regression and generalized estimating equation (GEE)

After the optimal trajectory model was selected, logistic regression was used to generate the AEH infection risk of different sexual behaviour trajectories. A generalized estimating equation (GEE) was constructed to compare the risk factors for sexual behaviour trajectories. The multivariable analysis included variables with 2-tailed $P$ < 0.1 in the univariate analysis.

#### Restricted cubic splines (RCS)

The association between the follow-up period and the risk of AEH infection was evaluated with restricted cubic splines (RCSs) based on the Cox proportional hazards model. Five knots were chosen at the 5th, 25th, 50th, 75th, and 95th percentiles of follow-up time. SAS 9.4 (SAS Institute Inc.) was used for statistical analyses.

## Results

### Sample characteristics

The demographic data and sexual behaviour at baseline are displayed in Table 1. During the period from October 2011 to December 2019, 1974 participants were followed for a total of 3633 person-years with 64 AEH infections documented. Participants were aged between 18 and 60, and most were under 50 years old (83%). Approximately half of the participants were married (53.55%) and lived in Tianjin (59.27%). The vast majority of participants declared that they had fewer than 10 sexual partners within the previous 6 months (86.95%) and fewer than 5 times of anal sex within the previous 7 days (97.82%). Less than half of the participants declared that they had used health services (46.48%) or undergone HIV testing (40.89%) in the last year.

There was no significant difference in demographic and sexual behaviour characteristics between the included and excluded participants (Additional file 2: Table S1).

**Table 1:** Baseline characteristics of study participants (N = 1974)

Variable	N (%)
Age	
< 30	756 (38.30)
30–49	883 (44.73)
≥ 50	335 (16.97)
Marital status	
Married	1056 (53.55)
Not married	916 (46.45)
Household registration location	
Local	1170 (59.27)
Nonlocal	804 (40.73)
Time of local residence	
≤ 1 year	450 (22.97)
More than 1 year	1509 (77.03)
Missing data	15
Working as a MSW	101 (5.12)
History of anal sex^a^	1834 (92.95)
Number of instances of anal sex^b^	
Less than 5 times	1795 (97.82)
5 times to 10 times	20 (1.09)
More than 10 times	20 (1.09)
Missing data	139
Condom use during last sexual intercourse with a man	1398 (71.80)
Missing data	27
Commercial sexual behaviour^a^	172 (8.77)
Missing data	13
Multiple sexual partners^a^	190 (12.56)
Missing data	461
Number of anal sex partners^a^	
≤ 10	873 (86.95)
> 10	131 (13.05)
Missing data	930
Health services received in the last year	917 (46.48)
Missing data	1
HIV testing in the last year	619 (40.89)
Missing data	460

^a^Within the previous 6 months

^b^Within the previous 7 days

### Sexual risk behaviour trajectories

**Fig. 1:**
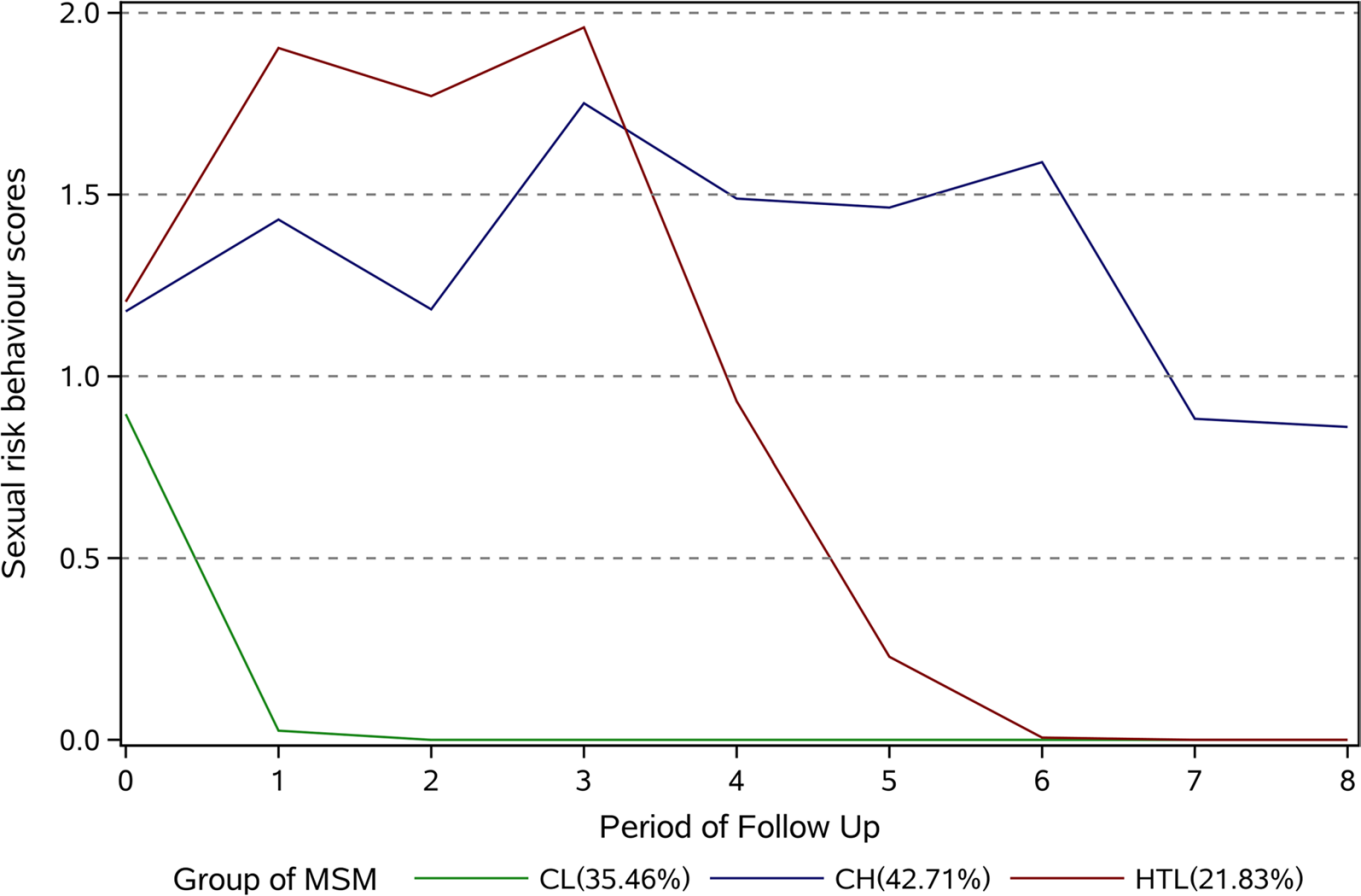
Sexual risk behaviour trajectories among MSM

As shown in Fig. 1, three sexual behaviour trajectories were identified: CL (consistently low risk), CH (consistently high risk) and HTL (high to low risk). The model fit process is displayed in Additional file 1: Tables S2–S7. The CL group (35.46%) was characterized by a group of men who observed the transition of risky behaviour to safe behaviour in the 1st year and then maintained safe behaviour. The CH group (42.71%) comprised a group of men with persistently risky sexual behaviour during follow-up. The HTL group (21.83%) consisted of a group of men who reported an increase in risky sexual behaviour during the first 3 years and then reported a rapid decrease.

### AEH infection of different trajectories

**Fig. 2:**
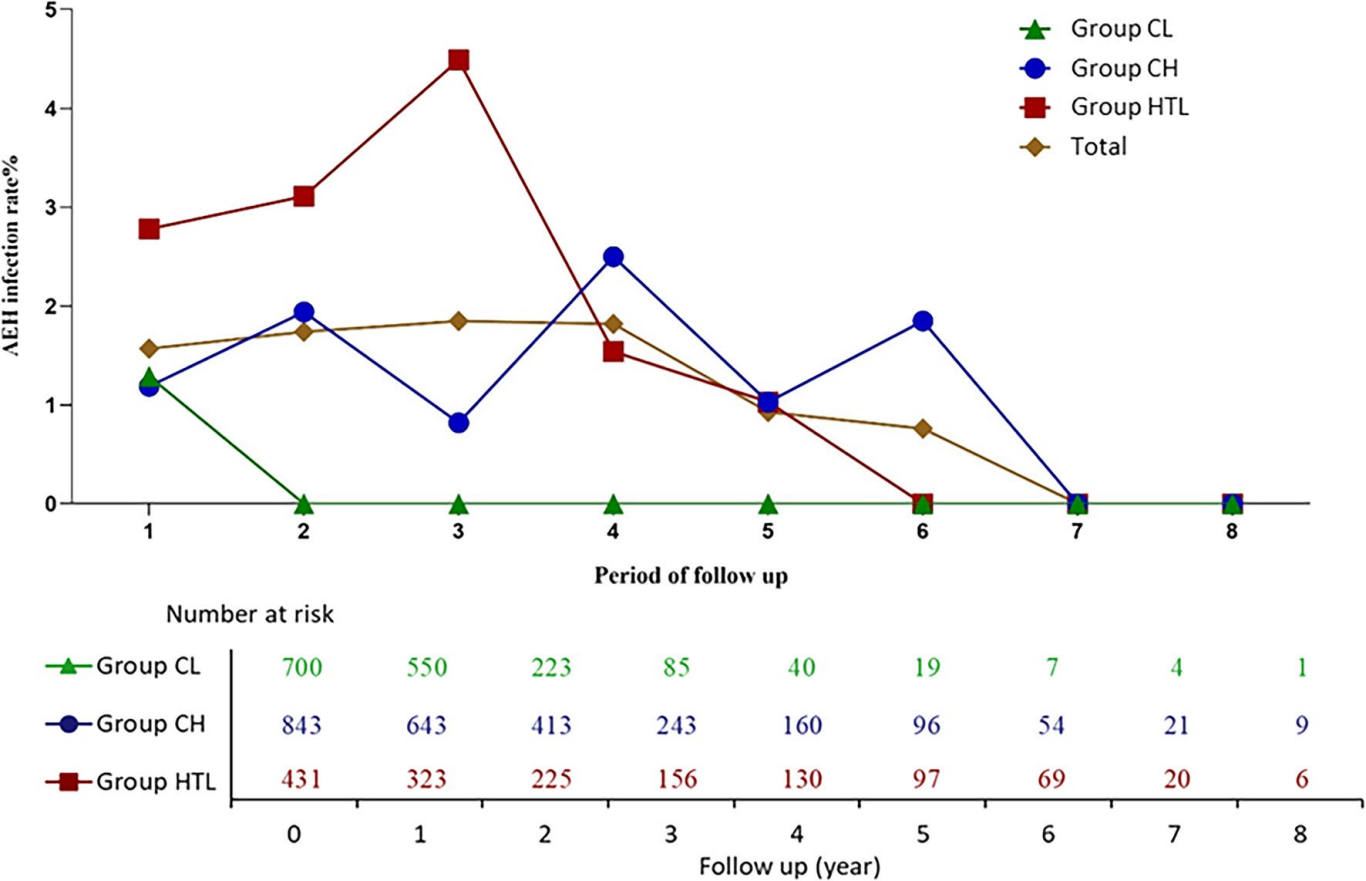
AEH infection rate of sexual risk behaviour trajectories over follow-up time

The AEH infection rate over the follow-up time of different trajectories and all participants is shown in Fig. 2. The AEH infection rate of the CL group remained at a low level at the 8-year follow-up, while the CH group fluctuated between 1% and 3%. The AEH infection in the first 3 years was largely attributed to the HTL group, which was consistent with the high risky behaviour score in the corresponding period of the HTL group.

Participants in the CH group and the HTL group had higher AEH infection rates than those in the CL group (3.08%, 6.73% and 1.28%, respectively) (Additional file 2: Table S2), and the ORs were 2.44 (1.14, 5.25) and 5.54 (2.60, 11.82), respectively (Table 2). The AEH acquisition risk among all participants over the follow-up period is shown in Additional file 3: Fig. S2.

**Table 2:** Differences in the risk of AEH infection between sexual risk behaviour trajectories among MSM

Trajectories	OR	95%CI	* P* value
CH-CL	2.44	1.14–5.25	0.02
HTL-CL	5.54	2.60–11.82	< 0.01
CH-HTL	0.44	0.26–0.76	0.89

### Characteristics of sexual risk behaviour trajectories

The baseline characteristics of participants at different trajectories are displayed in Additional file 2: Table S2. The average follow-up time for MSM in the CL group (1.19 years) was lower than that in the CH group (1.99 years) and the HTL group (2.61 years). The CL group and the CH group had a higher proportion of local registration (> 60%). The HTL group had a higher proportion of being nonlocal (53.13%), MSW (9.98%) and having undergone HIV testing last year (41.07%).

As shown in Table 3, compared with participants over 50 years old, MSM aged 30–50 years old were more likely to be in the CH group [aOR 1.54(1.09, 2.18)] and the HTL group [aOR 2.00(1.30, 3.07)]. MSM who underwent HIV testing in the last year also exhibited a higher probability of being in the CH group [aOR 1.79(1.31, 2.43)] and the HTL group [aOR 1.82(1.28, 2.57)]. MSM who received health services in the last year were less likely to be in the CH group [aOR 0.63(0.46, 0.85)]. The HTL group was characterized by a lower likelihood of being locally registered [OR 0.60(0.46, 0.79)], a higher likelihood of working as a MSW [aOR 4.15(2.04, 8.44)] and a higher likelihood of undergoing an HIV test in the last year [aOR 1.82(1.28, 2.57)].

**Table 3:** Differences in covariates of interest between sexual risk behaviour trajectories among MSM

Risk factors		Univariable analysis			Multivariable analysis		
		OR	95%CI	* P* value	aOR	95%CI	* P* value
Age							
CH							
< 30		1.53	1.19–1.95	< 0.001	1.13	0.71–1.77	0.610
30–49		1.44	1.09–1.91	0.01	1.54	1.09–2.18	**0.013**
HTL							
< 30		1.68	1.24–2.29	< 0.001	1.29	0.75–2.21	0.354
30–49		2.11	1.50–2.98	< 0.001	2.00	1.30–3.07	**0.002**
CL							
≥ 50		Ref.			Ref.		
Marital status							
CH							
Not married		0.79	0.63–0.97	0.028	0.91	0.66–1.24	0.535
HTL							
Not married		0.65	0.50–0.85	0.001	0.99	0.70–1.41	0.971
CL							
Married		Ref.			Ref.		
Household registration							
CH							
Local		0.88	0.71–1.10	0.267	1.03	0.76–1.40	0.854
HTL							
Local		0.60	0.46–0.79	< 0.001	1.04	0.7–1.49	0.824
CL							
Nonlocal		Ref.			Ref.		
Time of local residence							
CH							
≤ 1 year		1.01	0.78–1.30	0.958	0.99	0.69–1.40	0.935
HTL							
≤ 1 year		1.94	1.46–2.58	< 0.001	1.27	0.87–1.87	0.222
CL							
> 1 year		Ref.			Ref.		
Working as a MSW							
CH							
Yes		1.40	0.86–2.26	0.175	1.94	0.93–4.05	0.078
HTL							
Yes		3.02	1.89–4.82	< 0.001	4.15	2.04–8.44	**< 0.001**
CL							
No		Ref.			Ref.		
Health services received in the last year							
CH							
Yes		0.94	0.82–1.08	0.384	0.63	0.46–0.85	**0.003**
HTL							
Yes		1.17	0.99–1.37	0.053	0.74	0.52–1.04	0.0868
CL							
No		Ref.			Ref.		
HIV testing in the last year							
CH							
Yes		1.24	1.06–1.45	0.007	1.79	1.31–2.43	**< 0.001**
HTL							
Yes		1.60	1.35–1.91	< 0.001	1.82	1.28–2.57	**< 0.001**
CL							
No		Ref.			Ref.		

Variable with* P* < 0.05 is defined as significant (bold) for the mutivariable analysis

Multivariable GEE model adjusted for age, marital status, household registration, time of local residence, working as a MSW, health services received in the last year, HIV testing in the last year and AEH infection

## Discussion

Based on a VHCT project, our study found that the incidence of AEH infection among MSM was 1.76/100 person-years in 2011–2019, with 64 AEH infections documented in 3633 person-years of follow-up. In addition, our study found that MSM in the HTL and CH groups had a higher risk for AEH infection. In 2018, Amsterdam Cohort Studies [10] concluded that MSM in the falling high-risk group (corresponding to the HTL group in this study) were more susceptible to HIV infection than MSM in the low-risk group (corresponding to the CL group in this study), which is consistent with our results. Identifying the characteristics of CH and HTL groups is crucial for better implementing VHCT and exploring more comprehensive HIV intervention measures.

Our study found that the CH and HTL groups were more likely to be MSM aged between 30 and 50 years old. Previous studies about sexual risk behaviour trajectories have also found that high-risk groups possessed more younger MSM, among which Pines et al. inferred that a high-risk period of MSM occurred well beyond 30 years old [9, 11]. The above suggests that MSM under 50 years old were more likely to engage in high-risk sexual behaviour than MSM over 50 years old; therefore, VHCT should be performed more often among younger MSM.

Previous studies have suggested that repeated HIV-negative results may lead to reduced risk awareness among MSM, and thus, they may consider it unnecessary to engage in safe sex behaviour, thereby increasing the risk of HIV infection [20, 21]. Our study found that MSM in the CH group and the HTL group were more likely to be tested for HIV. In contrast, negative results on an HIV test may lead to an increase in risky behaviour, which could be interpreted by “risk compensation” [22]. In reality, although the VHCT included HIV counselling and HIV testing, the counselling part was sometimes ignored because the individuals had already received counselling or because the researchers were trying to administer tests efficiently. Therefore, HIV counselling should be given the same priority as HIV testing when implementing VHCT in the future.

The CH group was characterized by local registration and a lower probability of receiving health services in the last year. Poor access or reluctance to obtain health services due to stigma or worrying about discrimination may be the reason why MSM in the CH group maintained a high sexual risk behaviour score during follow-up. PrEP has been proven to be safe and highly effective in reducing HIV infection among MSM [23, 24]. Therefore, VHCT combined with PrEP may have a better preventive effect on AEH infection among the CH group.

Our study showed that the high AEH infection rate in the first 3 years was mainly attributed to the HTL group. MSM in the HTL group were characterized by being nonlocal, working as a MSW and having a high frequency of HIV testing. Previous studies have found that although most MSWs were poorly educated, they had a high rate of condom use with clients (53–99%) [25, 26]. MSM in the HTL group may be MSWs with high mobility who have high safety awareness. The high frequency of HIV testing among the HTL group may have led to the high rate of AEH infection detection. This result demonstrated that the VHCT can be convenient for MSWs and help to identify AEH infections in a timely manner.

The characteristics of this group and theoretical model of behaviour change may explain the trends of risk behaviour score and AEH infection rate of the HTL group. First, due to the high rate of AEH infection in the first 3 years, MSWs in the same circle may be afraid of being HIV infected, which reduces their risky behaviour. Second, the implementation of VHCT may raise the overall risk awareness in the MSM community, which indirectly improves the health situation of MSWs. Third, Alexander believed that people’s decision to initiate a new behaviour depended on their expectations for future results, while the decision to maintain one depended on their perceived satisfaction with the results they received. Moreover, behaviour change motivated by a desire to achieve a favourable state was more easily initiated than that motivated by a desire to avoid a negative state [27]. VHCT tends to identify potential people living with HIV; therefore, MSM may have difficulty initiating behaviour change due to the desire to avoid unknown or uninfected HIV status. However, given the low expectations, MSM are more likely to reap the benefits of healthy sex behaviour, and then a long-term behaviour change is sustained. In summary, the HTL trajectory indicates that encouraging MSM to set modest goals and reap the benefits of healthy sex may lead to better behaviour changes than constantly highlighting their potential susceptibility to sexually transmitted diseases (STDs) during HIV counselling.

The study had limitations. First, the study is based on observational data and lacks a control group, so the causal effect of VHCT on sexual behaviour change cannot be observed. A randomized control trial (RCT) is essential to test the effect of this intervention in the future. Second, a large proportion of MSM were lost to follow-up, especially after the 4th year. Therefore, the risk score and AEH infection rate for the long-term follow-up may be underestimated. VHCT has a limited effect on compliance, and VHCT combined with PrEP may have a better impact on improving the retention rate and reducing AEH infection.

Despite some limitations, our study has several strengths. First, the long follow-up time provided real-world data on VHCT, which could better guide the implementation of VHCT in the real world. Second, identifying distinct characteristics of MSM in high-risk behaviour trajectories was beneficial for developing precise prevention strategies.

## Conclusion

The study identified three sexual risk behaviour trajectories: CL, CH and HTL. The high AEH infection rate was attributed to the CH group and the HTL group. VHCT can provide convenience for MSM and help to identify AEH infection in a timely manner. VHCT should be performed more often among younger MSM, and HIV counselling should be given the same priority as HIV testing in the future. VHCT combined with PrEP may have a better prevention impact on MSM with a high risk of AEH infection.

## Supplementary Information

**Additional file 1.** Sexual risk behaviour score and fitting results of sexual risk behaviour trajectories. **Table S1.** Sexual risk behaviour score. **Table S2.** Five subgroups (3 3 3 3 3). **Table S3.** Five subgroups (3 1 3 1 1). **Table S4.** Four subgroups (3 3 3 3). **Table S5.** Four subgroups (1 1 1 3). **Table S6.** Three subgroups (3 3 3). **Table S7.** Three subgroups (3 1 2) (used in the main document)

**Additional file 2.** Baseline characteristics between included and excluded participants, between different sexual risk behaviour trajectories. **Table S1.** Baseline characteristics of included and excluded participants. **Table S2.** Baseline characteristics of sexual risk behaviour trajectories.

**Additional file 3.** Flow chart and restricted cubic splines (RCS) chart.

## Data Availability

The datasets analysed during the current study are available from the corresponding author on reasonable request.

## Abbreviations

HIV: Human immunodeficiency virus
MSM: Men who have sex with men
AEH: Acute and early HIV infection
VHCT: Voluntary HIV counselling and HIV testing
WB: Western blot
NAAT: Nucleic acid amplification test
CL: Consistently low risk
CH: Consistently high risk
HTL: High to low risk
MSW: Male sex worker
OR: Odds ratio
aOR: Adjusted odds ratio
PrEP: Preexposure prophylaxis
PEP: Postexposure prophylaxis
STDs: Sexually transmitted diseases
RCT: Randomized control trial
