# Supplementary material for: The risk of acute and early HIV (AEH) infection among MSM with different behaviour trajectories: an open cohort study in Tianjin, China, 2011–2019
Source: BMC Infect Dis. 2023 Jan 20;23:37. doi: 10.1186/s12879-023-08001-9 (PMC9862950; doi:10.1186/s12879-023-08001-9)
Supplement: Supplementary file 1 — Additional file 1. Sexual risk behaviour score and fitting results of sexual risk behaviour trajectories. Table S1. Sexual risk behaviour score. Table S2. Five subgroups (3 3 3 3 3). Table S3. Five subgroups (3 1 3 1 1). Table S4. Four subgroups (3 3 3 3). Table S5. Four subgroups (1 1 1 3). Table S6. Three subgroups (3 3 3). Table S7. Three subgroups (3 1 2) (used in the main document) [file 12879_2023_8001_MOESM1_ESM.pdf]

Additional file 1: Sexual risk behaviour score and fitting results of sexual risk behaviour trajectories

Sexual risk behaviour score

Table S1 Sexual risk behaviour score

Variable		Score
**Condom use during last sexual intercourse with a man**	Yes	0
	No	1
**Frequency of condom use**[†]	Seldom	2
	Sometimes	1
	Always	0
**Number of instances of anal sex within 7 days**	Less than 5 times	0
	5 times to 10 times	1
	More than 10 times	2
**Number of anal sex partners**[†]	≤ 10	0
	> 10	1

[†]within the previous 6 months

Fitting results of sexual risk behaviour trajectories

Table S2 Five subgroups (3 3 3 3 3)

Subgroup	Parameter	Estimate	$s_{\bar{x}}$	t	P value
**1**	Intercept	0.39170	0.16076	2.437	0.0149
	Linear	-8.81262	1.13655	-7.754	0.0000
	Quadratic	6.27995	0.74877	8.387	0.0000
	Cubic	-1.04891	0.12788	-8.203	0.0000
**2**	Intercept	0.57861	0.15971	3.623	0.0003
	Linear	4.08821	95.78189	0.043	0.9660
	Quadratic	-20.36571	52.65772	-0.387	0.6990
	Cubic	2.48239	6.75021	0.368	0.7131
**3**	Intercept	0.91557	0.07857	11.653	0.0000
	Linear	1.02104	0.13323	7.664	0.0000
	Quadratic	-0.40922	0.05580	-7.333	0.0000
	Cubic	0.03351	0.00586	5.718	0.0000
**4**	Intercept	0.68402	0.16146	4.236	0.0000
	Linear	9.62703	56.20621	0.171	0.8640
	Quadratic	-9.23708	63.23110	-0.146	0.8839
	Cubic	1.00260	7.02567	0.143	0.8865
**5**	Intercept	1.27171	0.18807	6.762	0.0000
	Linear	0.60289	0.31649	1.905	0.0568
	Quadratic	0.05497	0.14013	0.392	0.6948
	Cubic	-0.02416	0.01548	-1.561	0.1186
**BIC**	-8384.20	**AIC**	-8314.35	**LL**	-8289.35

Table S3 Five subgroups (3 1 3 1 1)

Subgroup	Parameter	Estimate	$s_{\bar{x}}$	t	P value
**1**	Intercept	0.30561	0.12515	2.442	0.0146
	Linear	-3.53521	7951000	0.000	1.0000
	Quadratic	-3.48732	25770000	0.000	1.0000
	Cubic	-3.55515	33610000	0.000	1.0000
**2**	Intercept	1.08492	0.25451	4.263	0.0000
	Linear	-0.00576	0.10379	-0.056	0.9557
**3**	Intercept	0.34227	0.27776	1.232	0.2179
	Linear	3.31334	164.88786	0.020	0.9840
	Quadratic	1.46920	184.33444	0.008	0.9936
	Cubic	-2.92560	198.61576	-0.015	0.9882
**4**	Intercept	1.05668	0.19691	5.366	0.0000
	Linear	-0.25330	0.12499	-2.027	0.0428
**5**	Intercept	1.37860	0.22625	6.093	0.0000
	Linear	0.41291	0.09971	4.141	0.0000
**BIC**	-8491.88	**AIC**	-8438.79	**LL**	-8419.79

Table S4 Four subgroups (3 3 3 3)

Subgroup	Parameter	Estimate	$s_{\bar{x}}$	*t*	*P* value
**1**	Intercept	0.41936	0.11587	3.619	0.0003
	Linear	-14.18229	1226.28487	-0.012	0.9908
	Quadratic	2.29128	282.69364	0.008	0.9935
	Cubic	-0.06429	17.09955	-0.004	0.9970
**2**	Intercept	0.74587	0.10902	6.841	0.0000
	Linear	0.01734	0.26206	0.066	0.9472
	Quadratic	-0.07376	0.10454	-0.706	0.4805
	Cubic	0.00865	0.00998	0.866	0.3864
**3**	Intercept	0.89798	0.13711	6.549	0.0000
	Linear	0.25128	0.43217	0.581	0.5610
	Quadratic	0.26721	0.31845	0.839	0.4014
	Cubic	-0.09857	0.05515	-1.787	0.0739
**4**	Intercept	1.15274	0.16085	7.167	0.0000
	Linear	0.88784	0.27634	3.213	0.0013
	Quadratic	-0.12718	0.12640	-1.006	0.3144
	Cubic	-0.00289	0.01435	-0.201	0.8406
**BIC**	-8435.58	**AIC**	-8379.70	**LL**	-8359.70

Table S5 Four subgroups (1 1 1 3)

Subgroup	Parameter	Estimate	$s_{\bar{x}}$	*t*	*P* value
**1**	Intercept	0.95133	0.14003	6.794	0.0000
	Linear	-0.35379	0.05411	-6.538	0.0000
**2**	Intercept	1.07476	0.10992	9.778	0.0000
	Linear	-0.04420	0.04698	-0.941	0.3468
**3**	Intercept	1.34503	0.22170	6.067	0.0000
	Linear	0.40739	0.09785	4.163	0.0000
**4**	Intercept	0.25123	0.13924	1.804	0.0713
	Linear	-5.66785	16570000	0.000	1.0000
	Quadratic	-4.91756	17220000	0.000	1.0000
	Cubic	-0.09287	2515000	0.000	1.0000
**BIC**	-8494.17	**AIC**	-8455.05	**LL**	--8441.05

Table S6 Three subgroups (3 3 3)

Subgroup	Parameter	Estimate	$s_{\bar{x}}$	*t*	*P* value
**1**	Intercept	0.40589	0.11301	3.592	0.0003
	Linear	-7.59321	2.20459	-3.444	0.0006
	Quadratic	2.25242	0.66506	3.387	0.0007
	Cubic	-0.16169	0.05058	-3.197	0.0014
**2**	Intercept	0.76990	0.09947	7.740	0.0000
	Linear	0.06803	0.30371	0.224	0.8228
	Quadratic	0.09781	0.19962	0.490	0.6242
	Cubic	-0.04882	0.03336	-1.463	0.1434

**3**	Intercept	0.94883	0.09176	10.341	0.0000
	Linear	0.71552	0.16112	4.441	0.0000
	Quadratic	-0.17590	0.06334	-2.777	0.0055
	Cubic	0.00838	0.00628	1.335	0.1819
**BIC**	-8436.98	**AIC**	-8395.07	**LL**	-8380.07

Table S7 Three subgroups (3 1 2) (used in the main document)

**Subgroup**	**Parameter**	**Estimate**	$s_{\bar{x}}$	***t***	***P* value**
**1**	Intercept	0.39298	0.11148	3.525	0.0004
	Linear	-1.78024	42845.71112	0.000	1.0000
	Quadratic	-1.60567	65321.86924	0.000	1.0000
	Cubic	-0.64193	50468.56515	0.000	1.0000
**2**	Intercept	0.89225	0.11792	7.566	0.0000
	Linear	0.03656	0.03189	1.146	0.2517
**3**	Intercept	0.83883	0.13200	6.355	0.0000
	Linear	1.10087	0.17544	6.275	0.0000
	Quadratic	-0.31683	0.03871	-8.185	0.0000
**BIC**	-8449.23	**AIC**	-8415.71	**LL**	-8403.71
