# Supplementary material for: The risk of acute and early HIV (AEH) infection among MSM with different behaviour trajectories: an open cohort study in Tianjin, China, 2011–2019
Source: BMC Infect Dis. 2023 Jan 20;23:37. doi: 10.1186/s12879-023-08001-9 (PMC9862950; doi:10.1186/s12879-023-08001-9)
Supplement: Supplementary file 2 — Additional file 2. Baseline characteristics between included and excluded participants, between different sexual risk behaviour trajectories. Table S1. Baseline characteristics of included and excluded participants. Table S2. Baseline characteristics of sexual risk behaviour trajectories. [file 12879_2023_8001_MOESM2_ESM.pdf]

Additional file 2: Baseline characteristics between included and excluded participants, between different sexual risk behaviour trajectories

Baseline characteristics between included and excluded participants

Table S1 Baseline characteristics of included and excluded participants

	**Included (N = 1974)**	**Excluded[⁋] (N = 4159)**	χ^2	***P* value**
**Age**			3.07	0.22
<45	1462(74.06)	3146(75.66)		
45-59	460(23.30)	890(21.40)		
>=60	52(2.63)	122(2.93)		
**Marital status**			2.38	0.12
Married	1056(53.55)	2311(55.65)		
Not married	916(46.45)	1842(44.35)		
**History of anal sex[†]**			2.00	0.16
Yes	1834(92.95)	3764(91.92)		
No	139(7.05)	331(8.08)		
**Homosexual anal sex [†]**				
Yes	1834(92.95)	3822(91.92)	2.00	0.16
No	139(7.05)	336(8.08)		
**Multiple sexual partners[†]**			0.07	0.79
Yes	190(12.56)	415(12.83)		
No	1323(87.44)	2819(87.17)		
**Commercial sexual behaviour[†]**			1.61	0.20
Yes	172(8.77)	324(7.82)		
No	1789(91.23)	3819(92.18)		
**Health services received in the last year**	917(46.48)	1705(41.00)	16.43	<0.0001
**HIV testing in the last year**	619(40.89)	1134(35.05)	15.06	0.0001

[†]within the previous 6 months; [⁋]Excluded participants are comprised of those who had only onc visit record (N = 4096) and diagnosed with chronic HIV during follow-up period (N = 63)

Baseline characteristics between different sexual risk behaviour trajectories

Table S2 Baseline characteristics of sexual risk behaviour trajectories

	CL (N = 700)	CH (N = 843)	HTL (N = 431)
**Follow-up time (mean)**	1.19	1.99	2.61
**Age**			
<30	291(41.57)	337(39.98)	128(29.70)
30-49	260(37.14)	369(43.77)	254(58.93)
≥50	149(21.29)	137(16.25)	49(11.37)
**Marital status**			
Married	397(56.71)	451(53.50)	208(48.26)
Not married	303(43.29)	390(46.26)	223(51.74)
**Household registration**			
Local	448(64.00)	520(61.68)	202(46.87)
Nonlocal	252(36.00)	323(38.32)	229(53.13)
**Time of local residence**			
≤1 year	138(19.71)	176(20.88)	136(31.55)
>1 year	554(79.14)	662(78.53)	293(67.98)
**Working as a MSW**	22(3.14)	36(4.27)	43(9.98)
**Health services received in the last year**	356(50.86)	361(42.82)	200(46.40)
**HIV testing in the last year**	203(29.00)	239(28.35)	177(41.07)
**AEH infection**	9(1.29)	26(3.08)	29(6.73)
