# Supplementary material for: The risk of acute and early HIV (AEH) infection among MSM with different behaviour trajectories: an open cohort study in Tianjin, China, 2011–2019
Source: BMC Infect Dis. 2023 Jan 20;23:37. doi: 10.1186/s12879-023-08001-9 (PMC9862950; doi:10.1186/s12879-023-08001-9)
Supplement: Supplementary file 3 — Additional file 3. Flow chart and restricted cubic splines (RCS) chart. [file 12879_2023_8001_MOESM3_ESM.pdf]

Additional file 3: Flow chart and restricted cubic splines (RCS) chart

Flow chart

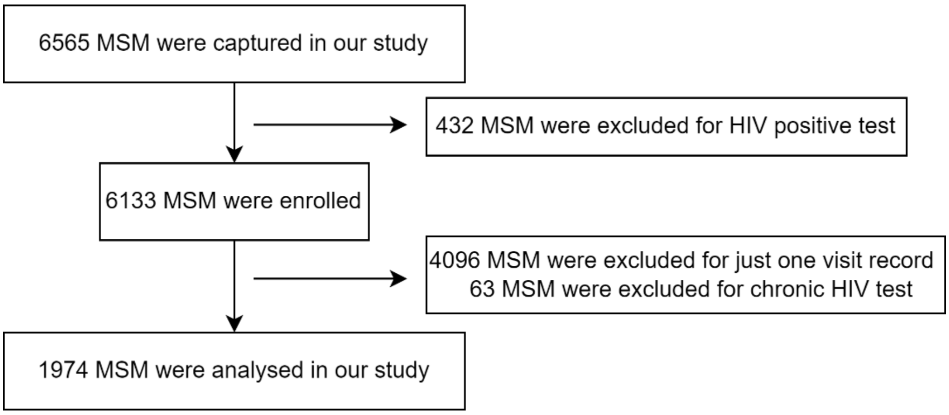

Fig. S1 Flow chart of the participants

Restricted cubic splines (RCS) chart

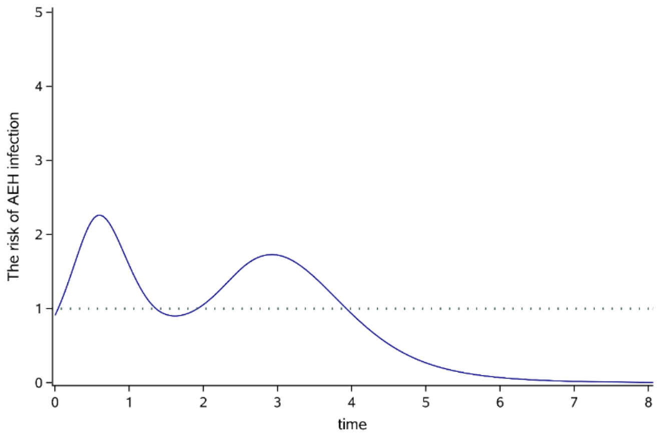

Fig. S2 Association between period of follow-up and the risk of AEH infection
